# 10-Hy­droxy­benzo[*h*]quinolinium tetra­chlorido(2-methyl­quinolin-8-olato-κ^2^ *N*,*O*)stannate(IV) methanol disolvate

**DOI:** 10.1107/S1600536811031461

**Published:** 2011-08-11

**Authors:** Ezzatollah Najafi, Marzieh Vafaee, Mostafa M. Amini, Seik Weng Ng

**Affiliations:** a Department of Chemistry, General Campus, Shahid Beheshti University, Tehran 1983963113, Iran; b Department of Chemistry, University of Malaya, 50603 Kuala Lumpur, Malaysia; c Chemistry Department, Faculty of Science, King Abdulaziz University, PO Box 80203 Jeddah, Saudi Arabia

## Abstract

In the disolvated title salt, (C_13_H_10_NO)[SnCl_4_(C_10_H_8_NO)]·2CH_3_OH, the Sn^IV^ atom is chelated by the *N*,*O*-bidentate 2-methyl­quinolin-8-olate ion and is further coordinated by four chloride ions, showing a distorted octa­hedral SnNOCl_4_ geometry. In the crystal, the cation and anion are linked to the methanol mol­ecules by O—H⋯O and N—H⋯O hydrogen bonds.

## Related literature

For the related compound, solvated 2-methyl-8-hy­droxy­quinolinium tetra­chlorido(quinolin-8-olato)stannate(IV), see: Vafaee *et al.* (2010 ▶).
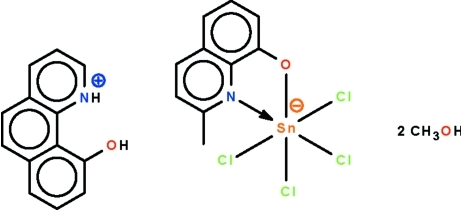


## Experimental

### 

#### Crystal data

(C_13_H_10_NO)[SnCl_4_(C_10_H_8_NO)]·2CH_4_O
*M* *_r_* = 678.97
Triclinic, 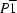

*a* = 7.5645 (2) Å
*b* = 10.1112 (3) Å
*c* = 17.7837 (5) Å
α = 98.105 (3)°
β = 95.653 (3)°
γ = 97.509 (3)°
*V* = 1325.56 (6) Å^3^
*Z* = 2
Mo *K*α radiation
μ = 1.40 mm^−1^
*T* = 100 K
0.30 × 0.30 × 0.10 mm

#### Data collection

Agilent SuperNova Dual diffractometer with an Atlas detector
Absorption correction: multi-scan (*CrysAlis PRO*; Agilent, 2010 ▶) *T*_min_ = 0.678, *T*_max_ = 0.873
10548 measured reflections
5871 independent reflections
5239 reflections with *I* > 2σ(*I*)
*R*_int_ = 0.056

#### Refinement

*R*[*F* ^2^ > 2σ(*F* ^2^)] = 0.041
*wR*(*F* ^2^) = 0.095
*S* = 1.11
5871 reflections
340 parameters
4 restraints
H atoms treated by a mixture of independent and constrained refinement
Δρ_max_ = 1.23 e Å^−3^
Δρ_min_ = −1.14 e Å^−3^

### 

Data collection: *CrysAlis PRO* (Agilent, 2010 ▶); cell refinement: *CrysAlis PRO*; data reduction: *CrysAlis PRO*; program(s) used to solve structure: *SHELXS97* (Sheldrick, 2008 ▶); program(s) used to refine structure: *SHELXL97* (Sheldrick, 2008 ▶); molecular graphics: *X-SEED* (Barbour, 2001 ▶); software used to prepare material for publication: *publCIF* (Westrip, 2010 ▶).

## Supplementary Material

Crystal structure: contains datablock(s) global, I. DOI: 10.1107/S1600536811031461/xu5283sup1.cif

Structure factors: contains datablock(s) I. DOI: 10.1107/S1600536811031461/xu5283Isup2.hkl

Additional supplementary materials:  crystallographic information; 3D view; checkCIF report

## Figures and Tables

**Table 1:** Hydrogen-bond geometry (Å, °)

*D*—H⋯*A*	*D*—H	H⋯*A*	*D*⋯*A*	*D*—H⋯*A*
O2—H2⋯O3	0.84 (1)	1.75 (2)	2.570 (3)	164 (5)
O3—H3⋯O1	0.84 (1)	1.94 (2)	2.746 (3)	162 (4)
N2—H1⋯O4	0.89 (1)	2.09 (3)	2.816 (4)	138 (3)

**Fig. 1.**
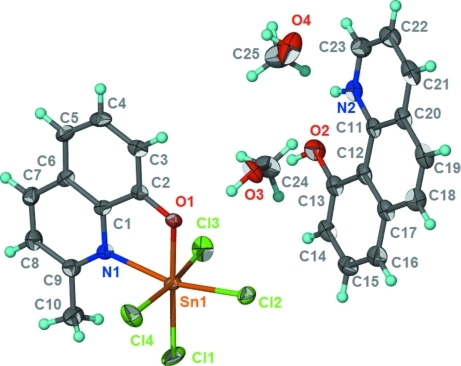
Thermal ellipsoid plot (Barbour, 2001) of (C14H10NO)[SnCl4(C10H8NO)].2CH3OH, at the 70% probability level; hydrogen atoms are drawn as spheres of arbitrary radius.

## supplementary crystallographic information

### Comment

We have been attempting to synthesize mixed-chelate tin(IV) compounds; in a recent study, we reacted stannic chloride with 8-hydroxyquinoline and 2-methyl-8-hydroxyquinoline (Vafaee *et al.*, 2010). However, the reaction yielded 2-methyl-8-hydroxyquinolinium tetrachlorido(quinolin-8-olato)stannate as an acetonitrile solvate. The ligand that enages in coordination is the one that is less sterically crowded. A similar synthesis but with 10-hydroxybenzo[*h*]quinoline and 2-methyl-8-hydroxyquinoline in methanol medium yielded the di-solvated title salt (Scheme I, Fig. 1). Similarly, the less sterically crowded ligand engages in chelation, so that the more crowded ligand is now protonated. The Sn^IV^ atom shows octahedral SnNOCl_4_ coordination. The cation and anion are linked to the methanol molecules by O–H···O and N–H···O hydrogen bonds. One of the solvent molecules functions only as acceptor whereas the other functions both as a donor as well as acceptor.

### Experimental

Stannic chloride pentahydrate (0.35 g, 1 mmol), 10-hydroxybenzo[*h*]quinoline (0.20 g, 1 mmol) and 2-methyl-8-hydroxyquinoline (0.16 g, 1 mmol) were loaded into a convection tube and the tube was filled with dry methanol and kept at 333 K. Yellow crystals were collected from the side arm after several days (in approximately yield 80%, m.p. 538 K).

### Refinement

Carbon-bound H-atoms were placed in calculated positions [C—H 0.95 to 0.98 Å, *U*_iso_(H) 1.2 to 1.5*U*_eq_(C)] and were included in the refinement in the riding model approximation.

The ammonium and hydroxy H-atoms were located in a difference Fourier map, and were refined with distance restraints of N–H 0.88±0.01, O–H 0.84±0.01 Å; their temperature factors were refined.

The final difference Fourier map had a peak as well as a hole in the vicinity ot Sn1.

Omitted from the refinement was the (0 0 2) reflection.

### Crystal data

**Table:**

(C_13_H_10_NO)[SnCl_4_(C_10_H_8_NO)]·2CH_4_O	*Z* = 2
*M**_r_* = 678.97	*F*(000) = 680
Triclinic, *P*1	*D*_x_ = 1.701 Mg m^−^^3^
Hall symbol: -P 1	Mo *K*α radiation, λ = 0.71073 Å
*a* = 7.5645 (2) Å	Cell parameters from 6565 reflections
*b* = 10.1112 (3) Å	θ = 2.3–29.3°
*c* = 17.7837 (5) Å	µ = 1.40 mm^−^^1^
α = 98.105 (3)°	*T* = 100 K
β = 95.653 (3)°	Prism, yellow
γ = 97.509 (3)°	0.30 × 0.30 × 0.10 mm
*V* = 1325.56 (6) Å^3^	

### Data collection

**Table:**

Agilent SuperNova Dual diffractometer with an Atlas detector	5871 independent reflections
Radiation source: SuperNova (Mo) X-ray Source	5239 reflections with *I* > 2σ(*I*)
Mirror	*R*_int_ = 0.056
Detector resolution: 10.4041 pixels mm^-1^	θ_max_ = 27.5°, θ_min_ = 2.5°
ω scans	*h* = −9→9
Absorption correction: multi-scan (*CrysAlis PRO*; Agilent, 2010)	*k* = −10→12
*T*_min_ = 0.678, *T*_max_ = 0.873	*l* = −22→23
10548 measured reflections	

### Refinement

**Table:**

Refinement on *F*^2^	Primary atom site location: structure-invariant direct methods
Least-squares matrix: full	Secondary atom site location: difference Fourier map
*R*[*F*^2^ > 2σ(*F*^2^)] = 0.041	Hydrogen site location: inferred from neighbouring sites
*wR*(*F*^2^) = 0.095	H atoms treated by a mixture of independent and constrained refinement
*S* = 1.11	*w* = 1/[σ^2^(*F*_o_^2^) + (0.0329*P*)^2^ + 1.5116*P*] where *P* = (*F*_o_^2^ + 2*F*_c_^2^)/3
5871 reflections	(Δ/σ)_max_ = 0.001
340 parameters	Δρ_max_ = 1.23 e Å^−^^3^
4 restraints	Δρ_min_ = −1.14 e Å^−^^3^

### Fractional atomic coordinates and isotropic or equivalent isotropic displacement parameters (Å^2^)

**Table:**

	*x*	*y*	*z*	*U*_iso_*/*U*_eq_	
Sn1	0.86883 (3)	0.09428 (2)	0.789295 (13)	0.01747 (8)	
Cl1	0.86363 (16)	−0.14417 (9)	0.78823 (6)	0.0417 (3)	
Cl2	0.63271 (12)	0.06051 (9)	0.68485 (5)	0.02469 (19)	
Cl3	0.66698 (12)	0.09658 (10)	0.88638 (5)	0.0303 (2)	
Cl4	1.10456 (13)	0.09761 (11)	0.70839 (5)	0.0340 (2)	
O1	0.8665 (3)	0.2980 (2)	0.78929 (13)	0.0164 (5)	
O2	0.3798 (4)	0.4948 (3)	0.64356 (15)	0.0274 (6)	
H2	0.453 (5)	0.464 (4)	0.672 (2)	0.041*	
O3	0.5605 (3)	0.4044 (3)	0.75020 (15)	0.0261 (6)	
H3	0.649 (4)	0.363 (4)	0.752 (3)	0.039*	
O4	0.4163 (4)	0.7760 (3)	0.73826 (19)	0.0459 (8)	
H4	0.507 (5)	0.836 (4)	0.749 (3)	0.069*	
N1	1.0833 (4)	0.1902 (3)	0.88721 (15)	0.0165 (6)	
N2	0.2612 (4)	0.6931 (3)	0.58555 (18)	0.0226 (6)	
H1	0.304 (5)	0.673 (4)	0.6302 (12)	0.027*	
C1	1.0870 (4)	0.3279 (3)	0.89787 (19)	0.0164 (7)	
C2	0.9706 (4)	0.3813 (3)	0.84513 (19)	0.0165 (7)	
C3	0.9725 (5)	0.5190 (3)	0.8541 (2)	0.0210 (7)	
H3A	0.8964	0.5564	0.8197	0.025*	
C4	1.0860 (5)	0.6053 (4)	0.9140 (2)	0.0240 (8)	
H4A	1.0843	0.6999	0.9191	0.029*	
C5	1.1982 (5)	0.5561 (4)	0.9646 (2)	0.0229 (7)	
H5	1.2740	0.6159	1.0043	0.028*	
C6	1.2003 (4)	0.4154 (3)	0.95736 (19)	0.0192 (7)	
C7	1.3109 (5)	0.3538 (4)	1.0069 (2)	0.0234 (8)	
H7	1.3881	0.4078	1.0484	0.028*	
C8	1.3067 (5)	0.2178 (4)	0.9950 (2)	0.0254 (8)	
H8	1.3821	0.1773	1.0281	0.031*	
C9	1.1914 (5)	0.1355 (4)	0.9339 (2)	0.0213 (7)	
C10	1.1918 (6)	−0.0137 (4)	0.9208 (2)	0.0320 (9)	
H10A	1.2077	−0.0433	0.8673	0.048*	
H10B	1.0775	−0.0596	0.9321	0.048*	
H10C	1.2907	−0.0360	0.9543	0.048*	
C11	0.2292 (4)	0.6061 (4)	0.5177 (2)	0.0189 (7)	
C12	0.2715 (4)	0.4711 (4)	0.5116 (2)	0.0202 (7)	
C13	0.3479 (4)	0.4138 (4)	0.5739 (2)	0.0215 (7)	
C14	0.3861 (5)	0.2837 (4)	0.5632 (2)	0.0245 (8)	
H14	0.4354	0.2465	0.6053	0.029*	
C15	0.3524 (5)	0.2065 (4)	0.4906 (2)	0.0256 (8)	
H15	0.3810	0.1171	0.4835	0.031*	
C16	0.2783 (5)	0.2574 (4)	0.4289 (2)	0.0256 (8)	
H16	0.2563	0.2032	0.3798	0.031*	
C17	0.2354 (4)	0.3891 (4)	0.4384 (2)	0.0214 (7)	
C18	0.1589 (5)	0.4437 (4)	0.3745 (2)	0.0263 (8)	
H18	0.1362	0.3889	0.3256	0.032*	
C19	0.1186 (5)	0.5699 (4)	0.3819 (2)	0.0262 (8)	
H19	0.0654	0.6017	0.3385	0.031*	
C20	0.1542 (4)	0.6568 (4)	0.4535 (2)	0.0222 (7)	
C21	0.1174 (5)	0.7900 (4)	0.4624 (2)	0.0295 (9)	
H21	0.0681	0.8246	0.4193	0.035*	
C22	0.1518 (5)	0.8716 (4)	0.5327 (2)	0.0288 (8)	
H22	0.1253	0.9615	0.5388	0.035*	
C23	0.2259 (5)	0.8189 (4)	0.5943 (2)	0.0290 (8)	
H23	0.2517	0.8735	0.6432	0.035*	
C24	0.4551 (6)	0.3602 (4)	0.8063 (2)	0.0329 (9)	
H24A	0.5270	0.3807	0.8566	0.049*	
H24B	0.4163	0.2626	0.7935	0.049*	
H24C	0.3496	0.4069	0.8075	0.049*	
C25	0.4131 (7)	0.7114 (5)	0.8025 (2)	0.0414 (10)	
H25A	0.5218	0.6691	0.8094	0.062*	
H25B	0.3070	0.6420	0.7955	0.062*	
H25C	0.4083	0.7777	0.8479	0.062*	

### Atomic displacement parameters (Å^2^)

**Table:**

	*U*^11^	*U*^22^	*U*^33^	*U*^12^	*U*^13^	*U*^23^
Sn1	0.02318 (13)	0.01235 (12)	0.01641 (13)	0.00327 (9)	−0.00053 (9)	0.00207 (8)
Cl1	0.0566 (7)	0.0134 (4)	0.0472 (6)	0.0041 (4)	−0.0256 (5)	0.0014 (4)
Cl2	0.0283 (4)	0.0200 (4)	0.0237 (4)	0.0035 (3)	−0.0062 (4)	0.0027 (3)
Cl3	0.0279 (5)	0.0373 (5)	0.0282 (5)	−0.0008 (4)	0.0084 (4)	0.0156 (4)
Cl4	0.0331 (5)	0.0530 (6)	0.0187 (4)	0.0224 (5)	0.0053 (4)	−0.0007 (4)
O1	0.0193 (11)	0.0139 (11)	0.0167 (11)	0.0049 (9)	0.0023 (9)	0.0027 (9)
O2	0.0339 (15)	0.0294 (15)	0.0213 (14)	0.0096 (12)	−0.0004 (12)	0.0102 (11)
O3	0.0230 (13)	0.0351 (15)	0.0266 (14)	0.0133 (11)	0.0089 (11)	0.0143 (11)
O4	0.0452 (18)	0.049 (2)	0.0390 (18)	−0.0141 (15)	−0.0075 (15)	0.0209 (15)
N1	0.0194 (13)	0.0164 (14)	0.0144 (14)	0.0025 (11)	0.0040 (11)	0.0031 (11)
N2	0.0192 (14)	0.0256 (16)	0.0242 (16)	0.0015 (13)	0.0018 (13)	0.0104 (13)
C1	0.0184 (15)	0.0135 (15)	0.0180 (16)	−0.0004 (13)	0.0074 (13)	0.0036 (12)
C2	0.0167 (15)	0.0177 (16)	0.0167 (16)	0.0029 (13)	0.0083 (13)	0.0039 (12)
C3	0.0242 (17)	0.0179 (17)	0.0240 (18)	0.0047 (14)	0.0099 (15)	0.0078 (14)
C4	0.0272 (18)	0.0135 (16)	0.032 (2)	0.0002 (14)	0.0135 (16)	0.0024 (14)
C5	0.0218 (17)	0.0196 (18)	0.0244 (19)	−0.0027 (14)	0.0071 (15)	−0.0042 (14)
C6	0.0171 (16)	0.0209 (17)	0.0186 (17)	−0.0020 (13)	0.0088 (14)	−0.0001 (13)
C7	0.0196 (17)	0.029 (2)	0.0192 (18)	0.0016 (15)	0.0024 (14)	−0.0036 (14)
C8	0.0246 (18)	0.029 (2)	0.0218 (18)	0.0049 (16)	−0.0029 (15)	0.0050 (15)
C9	0.0249 (17)	0.0204 (18)	0.0192 (17)	0.0047 (14)	0.0020 (14)	0.0047 (13)
C10	0.040 (2)	0.0217 (19)	0.032 (2)	0.0077 (17)	−0.0115 (18)	0.0049 (16)
C11	0.0128 (15)	0.0243 (18)	0.0214 (18)	0.0013 (13)	0.0046 (13)	0.0090 (14)
C12	0.0129 (15)	0.0285 (19)	0.0222 (18)	0.0034 (14)	0.0064 (14)	0.0109 (14)
C13	0.0182 (16)	0.0268 (19)	0.0217 (18)	0.0034 (14)	0.0051 (14)	0.0089 (14)
C14	0.0200 (17)	0.029 (2)	0.029 (2)	0.0058 (15)	0.0065 (15)	0.0139 (15)
C15	0.0227 (18)	0.0247 (19)	0.033 (2)	0.0053 (15)	0.0108 (16)	0.0089 (15)
C16	0.0238 (18)	0.031 (2)	0.0235 (19)	0.0039 (16)	0.0095 (15)	0.0048 (15)
C17	0.0166 (16)	0.0291 (19)	0.0215 (18)	0.0039 (14)	0.0079 (14)	0.0100 (14)
C18	0.0283 (19)	0.036 (2)	0.0170 (17)	0.0049 (16)	0.0064 (15)	0.0081 (15)
C19	0.0215 (17)	0.040 (2)	0.0220 (19)	0.0068 (16)	0.0067 (15)	0.0156 (16)
C20	0.0170 (16)	0.030 (2)	0.0236 (18)	0.0041 (14)	0.0071 (14)	0.0135 (15)
C21	0.0214 (18)	0.038 (2)	0.035 (2)	0.0083 (17)	0.0081 (17)	0.0216 (18)
C22	0.0288 (19)	0.027 (2)	0.034 (2)	0.0071 (16)	0.0080 (17)	0.0113 (16)
C23	0.0278 (19)	0.024 (2)	0.036 (2)	0.0037 (16)	0.0039 (17)	0.0075 (16)
C24	0.037 (2)	0.034 (2)	0.034 (2)	0.0086 (18)	0.0181 (18)	0.0113 (17)
C25	0.055 (3)	0.037 (2)	0.033 (2)	0.006 (2)	0.009 (2)	0.0052 (19)

### Geometric parameters (Å, °)

**Table:**

Sn1—O1	2.063 (2)	C9—C10	1.494 (5)
Sn1—N1	2.272 (3)	C10—H10A	0.9800
Sn1—Cl4	2.3988 (10)	C10—H10B	0.9800
Sn1—Cl2	2.4011 (8)	C10—H10C	0.9800
Sn1—Cl1	2.4036 (9)	C11—C20	1.414 (5)
Sn1—Cl3	2.4145 (9)	C11—C12	1.435 (5)
O1—C2	1.324 (4)	C12—C17	1.422 (5)
O2—C13	1.366 (4)	C12—C13	1.429 (5)
O2—H2	0.843 (10)	C13—C14	1.375 (5)
O3—C24	1.421 (5)	C14—C15	1.393 (5)
O3—H3	0.835 (10)	C14—H14	0.9500
O4—C25	1.395 (5)	C15—C16	1.376 (5)
O4—H4	0.838 (10)	C15—H15	0.9500
N1—C9	1.333 (4)	C16—C17	1.403 (5)
N1—C1	1.374 (4)	C16—H16	0.9500
N2—C23	1.325 (5)	C17—C18	1.436 (5)
N2—C11	1.369 (5)	C18—C19	1.342 (5)
N2—H1	0.888 (10)	C18—H18	0.9500
C1—C6	1.413 (5)	C19—C20	1.423 (5)
C1—C2	1.433 (5)	C19—H19	0.9500
C2—C3	1.377 (5)	C20—C21	1.401 (5)
C3—C4	1.412 (5)	C21—C22	1.380 (6)
C3—H3A	0.9500	C21—H21	0.9500
C4—C5	1.366 (5)	C22—C23	1.385 (5)
C4—H4A	0.9500	C22—H22	0.9500
C5—C6	1.413 (5)	C23—H23	0.9500
C5—H5	0.9500	C24—H24A	0.9800
C6—C7	1.418 (5)	C24—H24B	0.9800
C7—C8	1.358 (5)	C24—H24C	0.9800
C7—H7	0.9500	C25—H25A	0.9800
C8—C9	1.415 (5)	C25—H25B	0.9800
C8—H8	0.9500	C25—H25C	0.9800
			
O1—Sn1—N1	77.28 (9)	C9—C10—H10C	109.5
O1—Sn1—Cl4	90.31 (7)	H10A—C10—H10C	109.5
N1—Sn1—Cl4	86.85 (7)	H10B—C10—H10C	109.5
O1—Sn1—Cl2	86.01 (6)	N2—C11—C20	116.6 (3)
N1—Sn1—Cl2	163.26 (7)	N2—C11—C12	121.9 (3)
Cl4—Sn1—Cl2	94.22 (3)	C20—C11—C12	121.5 (3)
O1—Sn1—Cl1	178.56 (7)	C17—C12—C13	117.9 (3)
N1—Sn1—Cl1	103.94 (7)	C17—C12—C11	117.7 (3)
Cl4—Sn1—Cl1	90.53 (4)	C13—C12—C11	124.4 (3)
Cl2—Sn1—Cl1	92.77 (3)	O2—C13—C14	122.6 (3)
O1—Sn1—Cl3	90.14 (7)	O2—C13—C12	116.5 (3)
N1—Sn1—Cl3	84.93 (7)	C14—C13—C12	120.8 (3)
Cl4—Sn1—Cl3	171.46 (3)	C13—C14—C15	120.0 (3)
Cl2—Sn1—Cl3	94.32 (3)	C13—C14—H14	120.0
Cl1—Sn1—Cl3	89.20 (4)	C15—C14—H14	120.0
C2—O1—Sn1	116.63 (19)	C16—C15—C14	121.1 (3)
C13—O2—H2	109 (3)	C16—C15—H15	119.5
C24—O3—H3	105 (3)	C14—C15—H15	119.5
C25—O4—H4	104 (4)	C15—C16—C17	120.2 (3)
C9—N1—C1	119.5 (3)	C15—C16—H16	119.9
C9—N1—Sn1	131.3 (2)	C17—C16—H16	119.9
C1—N1—Sn1	109.2 (2)	C16—C17—C12	119.9 (3)
C23—N2—C11	124.4 (3)	C16—C17—C18	120.7 (3)
C23—N2—H1	110 (3)	C12—C17—C18	119.3 (3)
C11—N2—H1	126 (3)	C19—C18—C17	121.8 (3)
N1—C1—C6	122.7 (3)	C19—C18—H18	119.1
N1—C1—C2	117.1 (3)	C17—C18—H18	119.1
C6—C1—C2	120.3 (3)	C18—C19—C20	121.1 (3)
O1—C2—C3	122.2 (3)	C18—C19—H19	119.4
O1—C2—C1	119.5 (3)	C20—C19—H19	119.4
C3—C2—C1	118.3 (3)	C21—C20—C11	119.3 (3)
C2—C3—C4	120.8 (3)	C21—C20—C19	122.2 (3)
C2—C3—H3A	119.6	C11—C20—C19	118.5 (3)
C4—C3—H3A	119.6	C22—C21—C20	121.0 (4)
C5—C4—C3	121.7 (3)	C22—C21—H21	119.5
C5—C4—H4A	119.2	C20—C21—H21	119.5
C3—C4—H4A	119.2	C21—C22—C23	118.2 (4)
C4—C5—C6	119.3 (3)	C21—C22—H22	120.9
C4—C5—H5	120.3	C23—C22—H22	120.9
C6—C5—H5	120.3	N2—C23—C22	120.6 (4)
C1—C6—C5	119.6 (3)	N2—C23—H23	119.7
C1—C6—C7	116.4 (3)	C22—C23—H23	119.7
C5—C6—C7	123.9 (3)	O3—C24—H24A	109.5
C8—C7—C6	120.1 (3)	O3—C24—H24B	109.5
C8—C7—H7	120.0	H24A—C24—H24B	109.5
C6—C7—H7	120.0	O3—C24—H24C	109.5
C7—C8—C9	120.8 (3)	H24A—C24—H24C	109.5
C7—C8—H8	119.6	H24B—C24—H24C	109.5
C9—C8—H8	119.6	O4—C25—H25A	109.5
N1—C9—C8	120.5 (3)	O4—C25—H25B	109.5
N1—C9—C10	119.5 (3)	H25A—C25—H25B	109.5
C8—C9—C10	119.9 (3)	O4—C25—H25C	109.5
C9—C10—H10A	109.5	H25A—C25—H25C	109.5
C9—C10—H10B	109.5	H25B—C25—H25C	109.5
H10A—C10—H10B	109.5		
			
N1—Sn1—O1—C2	−5.0 (2)	C1—N1—C9—C10	−178.0 (3)
Cl4—Sn1—O1—C2	−91.7 (2)	Sn1—N1—C9—C10	5.3 (5)
Cl2—Sn1—O1—C2	174.1 (2)	C7—C8—C9—N1	−0.7 (6)
Cl3—Sn1—O1—C2	79.8 (2)	C7—C8—C9—C10	178.6 (4)
O1—Sn1—N1—C9	−178.4 (3)	C23—N2—C11—C20	0.3 (5)
Cl4—Sn1—N1—C9	−87.4 (3)	C23—N2—C11—C12	179.5 (3)
Cl2—Sn1—N1—C9	178.4 (2)	N2—C11—C12—C17	−179.2 (3)
Cl1—Sn1—N1—C9	2.4 (3)	C20—C11—C12—C17	0.0 (5)
Cl3—Sn1—N1—C9	90.3 (3)	N2—C11—C12—C13	0.6 (5)
O1—Sn1—N1—C1	4.6 (2)	C20—C11—C12—C13	179.8 (3)
Cl4—Sn1—N1—C1	95.7 (2)	C17—C12—C13—O2	−179.5 (3)
Cl2—Sn1—N1—C1	1.5 (4)	C11—C12—C13—O2	0.7 (5)
Cl1—Sn1—N1—C1	−174.57 (19)	C17—C12—C13—C14	0.3 (5)
Cl3—Sn1—N1—C1	−86.7 (2)	C11—C12—C13—C14	−179.4 (3)
C9—N1—C1—C6	−0.7 (5)	O2—C13—C14—C15	−179.3 (3)
Sn1—N1—C1—C6	176.6 (3)	C12—C13—C14—C15	0.8 (5)
C9—N1—C1—C2	178.8 (3)	C13—C14—C15—C16	−1.0 (5)
Sn1—N1—C1—C2	−3.8 (3)	C14—C15—C16—C17	0.0 (5)
Sn1—O1—C2—C3	−175.9 (2)	C15—C16—C17—C12	1.2 (5)
Sn1—O1—C2—C1	4.7 (4)	C15—C16—C17—C18	179.7 (3)
N1—C1—C2—O1	−0.2 (4)	C13—C12—C17—C16	−1.3 (5)
C6—C1—C2—O1	179.4 (3)	C11—C12—C17—C16	178.4 (3)
N1—C1—C2—C3	−179.6 (3)	C13—C12—C17—C18	−179.9 (3)
C6—C1—C2—C3	0.0 (5)	C11—C12—C17—C18	−0.1 (5)
O1—C2—C3—C4	−179.5 (3)	C16—C17—C18—C19	−179.2 (3)
C1—C2—C3—C4	−0.2 (5)	C12—C17—C18—C19	−0.7 (5)
C2—C3—C4—C5	0.3 (5)	C17—C18—C19—C20	1.5 (6)
C3—C4—C5—C6	−0.3 (5)	N2—C11—C20—C21	0.1 (5)
N1—C1—C6—C5	179.5 (3)	C12—C11—C20—C21	−179.2 (3)
C2—C1—C6—C5	0.0 (5)	N2—C11—C20—C19	−179.9 (3)
N1—C1—C6—C7	−0.5 (5)	C12—C11—C20—C19	0.8 (5)
C2—C1—C6—C7	180.0 (3)	C18—C19—C20—C21	178.4 (3)
C4—C5—C6—C1	0.2 (5)	C18—C19—C20—C11	−1.6 (5)
C4—C5—C6—C7	−179.8 (3)	C11—C20—C21—C22	−0.6 (5)
C1—C6—C7—C8	1.2 (5)	C19—C20—C21—C22	179.4 (4)
C5—C6—C7—C8	−178.9 (3)	C20—C21—C22—C23	0.9 (6)
C6—C7—C8—C9	−0.6 (5)	C11—N2—C23—C22	0.0 (6)
C1—N1—C9—C8	1.3 (5)	C21—C22—C23—N2	−0.5 (6)
Sn1—N1—C9—C8	−175.4 (2)		

### Hydrogen-bond geometry (Å, °)

**Table:**

*D*—H···*A*	*D*—H	H···*A*	*D*···*A*	*D*—H···*A*
O2—H2···O3	0.84 (1)	1.75 (2)	2.570 (3)	164 (5)
O3—H3···O1	0.84 (1)	1.94 (2)	2.746 (3)	162 (4)
N2—H1···O4	0.89 (1)	2.09 (3)	2.816 (4)	138 (3)

## References

[bb1] Agilent (2010). *CrysAlis PRO* Agilent Technologies, Yarnton, England.

[bb2] Barbour, L. J. (2001). *J. Supramol. Chem.* **1**, 189–191.

[bb3] Sheldrick, G. M. (2008). *Acta Cryst.* A**64**, 112–122. 10.1107/S0108767307043930 18156677

[bb4] Vafaee, M., Mohammadnezhad, G., Amini, M. M. & Ng, S. W. (2010). *Acta Cryst.* E**66**, m381–m382. 10.1107/S160053681000810X PMC2983816 21580491

[bb5] Westrip, S. P. (2010). *J. Appl. Cryst.* **43**, 920–925.

